# Effect of Ion Beam Balancing on Frequency Split for Fourth Harmonic Mass Defect of Hemispherical Resonator

**DOI:** 10.3390/s25185888

**Published:** 2025-09-20

**Authors:** Boran Li, Changhong Wang, Zhen Fang

**Affiliations:** 1 Space Control and Inertial Technology Research Center, Harbin Institute of Technology, Harbin 150001, China; scit201@163.com; 2 The 26th Institute of China Electronics Technology Group Corporation, Chongqing 400600, China; hrg@sipat.com

**Keywords:** hemispherical resonator, frequency split, mass balancing

## Abstract

Based on the energy equation of the hemispherical resonator, this study analyzes the influence of ion beam balancing on the frequency split of the hemispherical resonator. Firstly, the formula for mass defects and the resonant frequency of the resonator is obtained through the energy equation. All mass defects of the resonator are equivalent to fourth harmonic wall thickness defects. The quantitative relationship between harmonic wall thickness defects and the resonant frequency and the geometric relationship between the heavy axis of the resonator and the distribution of mass defects are determined. Secondly, the balancing function is introduced into the resonant frequency equation of the hemispherical resonator, and a mathematical model is established for the resonant frequency, defect wall thickness, and balancing depth of the hemispherical resonator. By introducing relevant errors, the impact of balancing errors on the frequency characteristics of the hemispherical resonator is calculated. Finally, an ion beam balancing experiment is designed to verify the effectiveness of the theory. The results show that the frequency split can be better than 0.001 Hz after balancing, effectively improving the hemispherical resonator’s performance.

## 1. Introduction

Hemispherical resonator gyroscopes, as newly developed solid-state vibratory gyros, have the advantages of high precision, a compact size, and low failure rates. Their unique advantage of continuous long-term operation makes them widely applicable in fields such as aviation, aerospace, and naval equipment. There are a lot of theories and experiments that have been performed to analyze the mechanism of the resonator. Lynch established the generalized equations of motion of CVG, which considered the frequency split and damping non-uniformity [1]. Choi and Kim pointed out that the effect of point masses on the hemispherical shell could be described by a function including the angle of the vibration mode and frequency split [2,3]. Equating mass defects to density defects and expanding them in the form of harmonic components along the ring, Huo et al. established the equations of motion of a resonator and analyzed the effect of mass harmonic errors on frequency splitting and standing wave drift [4,5]. Matveev et al. derived a model for the relation between mass error and frequency splitting, which can be used to analyze the necessity of balancing the defective mass [6]. Based on the Restoring Force Surface method and the Chebyshev polynomials, Mohammad et al. presented a data-driven nonparametric identification method of an MEMS resonator using only experimental data. This approach can be applied to micro structures, which are naturally curved due to fabrication imperfections [7,8]. All of these theoretical analyses and studies have important implications and engineering applications for frequency splitting modification.

As the most important component of a hemispherical resonant gyroscope, the hemispherical resonator always has varying degrees of uneven wall thickness and structural damage due to limitations in processing accuracy and technology. In order to improve the accuracy of the hemispherical resonant gyroscope, it is necessary to physically adjust the processed hemispherical resonator to achieve the ideal vibration form as much as possible. In practice, the major frequency split balancing methods include mechanical trimming, laser ablation, chemical etching, and focus ion beam etching. Wang et al. reported a method for reducing the frequency splitting using a directional grinding technique [9]. Raspopov et al. used a scribing needle to remove a certain mass along the heavy axis of the resonator [10]. Zhao et al. employed a balancing method using femtosecond laser ablation, and the experimental results showed that the frequency split was reduced to 0.008 Hz [11]. Basarab et al. designed a procedure for removing the defective mass through a chemical etching process, and mainly discussed the angle of resonator rotation about the axis of symmetry, inclination, depth of the spherical shell’s immersion into the chemical bath, and etching time [12]. Russian company Medicon has developed an ion beam trimming device specifically for hemispherical resonators, achieving a frequency split detection accuracy of 0.2 mHz [13]. However, the laser ablation and mechanical trimming can easily cause a decrease in quality factor [14,15,16,17,18]. Chemical etching only can be regarded as a medium-precision trimming method that is sensitive to the temperature stability and solution concentration [19,20,21]. Finally, focus ion beam etching, which has a more comprehensive frequency balancing range, better robustness, and stability, has become a high-precision balancing method [22,23].

The mentioned theories and studies provide a basic understanding of the frequency tuning mechanism. However, there are still some shortcomings, which are reflected in the following aspects.

(1) Some studies describe mass defects using the mass point. This can pose difficulties in identifying the main sources of error that cause frequency splitting.
(2) The mass imperfection is expressed in terms of density. With advancements in production equipment and improvements in processing technology, the risk of harmonic-type mass defects being formed by density is becoming smaller. The main reason for this is the uneven wall thickness caused by a low processing accuracy and process defects. Using density to describe quality defects is not sufficient in physical terms.
(3) Research and experiments largely focus on the reduction effect of frequency splitting, with less attention given to the impact of balancing errors. This raises the question of whether frequency splitting can be fully minimized to zero if there are errors in balancing.

Based on the energy model of the hemispherical resonator, this article establishes a frequency model for the mass defects. By introducing the balancing function, the changes in the frequency split and the axis position during the balancing process are calculated. Then, an ion beam trimming process based on the ion beam etching technique is designed to verify the theoretical effectiveness. The structure of the paper is arranged as follows: In Section 2, a hemispherical resonator frequency model with the fourth harmonic of the mass defect based on resonator energy equations is developed, which reveals the numerical relationship between the thickness of mass defects and frequency splitting. In Section 3, after incorporating the balancing function into the frequency model, the effectiveness of trimming the frequency splitting with the balancing position, depth, and errors is calculated. In Section 4, based on the theoretical results, a focused ion beam balancing process is designed to remove the equivalent defect mass from the heavy axis of the resonator, and the experimental results are analyzed and discussed. Finally, the conclusion is presented in Section 5.

## 2. Energy Equation and Frequency Split of Resonator

According to the theory of elastic thin shells, the elastic potential energy of the hemispherical resonator is as follows:

(1) $$U=\frac{1}{2}\int_{\varphi_{0}}^{\varphi_{F}}\int_{0}^{2\pi }\frac{E}{\left(1-\mu^{2}\right)}\left[\varepsilon_{1}^{2}+\varepsilon_{2}^{2}+2\mu \varepsilon_{1}\varepsilon_{2}+\frac{1-\mu }{2}\varepsilon_{12}^{2}\right.\left.+\frac{h^{2}}{12}\left(\lambda_{1}^{2}+\lambda_{2}^{2}+2\mu \lambda_{1}\lambda_{2}+\frac{1-\mu }{2}\lambda_{12}^{2}\right)\right]hd\theta d\varphi$$

where $\varphi_{0}$ is the bottom angle of the resonator, $\varphi_{F}$ is the top angle of the resonator, *E* is the elastic modulus, *μ* is the material’s Poisson’s ratio, *h* is the wall thickness of the hemispherical shell, and the midplane strain and the midplane bending deformation of the stretchable hemispherical thin shell are as follows:

(2) $$\left\{\begin{array}{l} \varepsilon_{1}=\frac{\left(\frac{\partial u}{\partial \varphi }+w\right)}{R}\\ \varepsilon_{2}=\frac{\left(\frac{\partial v}{\partial \theta }\frac{1}{\sin\varphi }+\frac{vcos\varphi }{\sin\varphi }+w\right)}{R}\\ \varepsilon_{12}=\frac{\left(\frac{\partial u}{\partial \theta }\frac{1}{\sin\varphi }+\frac{\partial v}{\partial \varphi }-\frac{v\cos\varphi }{\sin\varphi }\right)}{R} \end{array}\right.$$

(3) $$\left\{\begin{array}{l} \lambda_{1}=\left(-\frac{\partial^{2}w}{\partial \varphi^{2}}+\frac{\partial u}{\partial \varphi }\right)/R^{2}\\ \lambda_{2}=\left(-\frac{\partial^{2}w}{\partial \theta^{2}}\frac{1}{\sin^{2}\varphi }-\frac{\cos\varphi }{\sin\varphi }\frac{\partial w}{\partial \varphi }+\frac{u\cos\varphi }{\sin\varphi }+\frac{\partial v}{\partial \theta }\frac{1}{\sin\varphi }\right)/R^{2}\\ \lambda_{12}=\left[\frac{\partial u}{\partial \theta }\frac{1}{\sin\varphi }+\frac{\partial v}{\partial \varphi }-\frac{v\cos\varphi }{\sin\varphi }+2\left(\frac{\cos\varphi }{\sin^{2}\varphi }\frac{\partial w}{\partial \theta }-\frac{\partial^{2}w}{\partial \varphi \partial \theta }\frac{1}{\sin\varphi }\right)\right]/R^{2} \end{array}\right.$$

in which *u*, *v*, and *w* are the displacement components of the shell, tangent, and radial, and *R* is the mid-surface radius of the resonator.

The kinetic energy of the shell is

(4) $$T=\frac{1}{2}\int_{\varphi_{0}}^{\varphi_{F}}\int_{0}^{2\pi }\left[{\left(\frac{\partial u}{\partial t}\right)}^{2}+{\left(\frac{\partial v}{\partial t}\right)}^{2}+{\left(\frac{\partial w}{\partial t}\right)}^{2}\right]h\rho d\theta d\varphi$$

where ρ is the density of the resonator.

Using $T_{\max}=U_{\max}$, the following can be concluded:

(5) $$\omega^{2}=\frac{K}{m}$$

(6) $$K=\int_{\varphi_{0}}^{\varphi_{F}}\int_{0}^{2\pi }\frac{E}{\left(1+\mu \right)R^{4}}\frac{n^{2}{\left(n^{2}-1\right)}^{2}}{6\sin^{3}\varphi }\tan^{2n}\frac{\varphi }{2}h^{3}d\theta d\varphi$$

(7) $$m=\int_{\varphi_{0}}^{\varphi_{F}}\int_{0}^{2\pi }\left[\sin^{2}\varphi +{\left(n+\cos\varphi \right)}^{2}cos^{2}n\left(\theta +\Psi \right)\right]\tan^{2n}\frac{\varphi }{2}\sin\varphi h\rho d\theta d\varphi$$

in which n is the circumferential wavenumber of the resonant mode, and Ψ is the nodal deflection angle of the resonator’s standing wave.

For m, after transformation, it can be converted to

(8) $$m=m_{01}+\frac{1}{2}m_{02}\cos2n\Psi -\frac{1}{2}m_{03}\sin2n\Psi$$

where $m_{01}$, $m_{02}$, and $m_{03}$ are constants to be determined, and

$$m_{01}=\int_{\varphi_{0}}^{\varphi_{F}}\int_{0}^{2\pi }\left[\sin^{2}\varphi +\frac{1}{2}{\left(n+\cos\varphi \right)}^{2}\right]\tan^{2n}\frac{\varphi }{2}\sin\varphi h\rho d\theta d\varphi$$

$$m_{02}=\int_{\varphi_{0}}^{\varphi_{F}}\int_{0}^{2\pi }{\left(n+\cos\varphi \right)}^{2}\tan^{2n}\frac{\varphi }{2}\sin\varphi \cos2n\theta h\rho d\theta d\varphi$$

$$m_{03}=\int_{\varphi_{0}}^{\varphi_{F}}\int_{0}^{2\pi }{\left(n+\cos\varphi \right)}^{2}tan^{2n}\frac{\varphi }{2}\sin\varphi \sin2n\theta h\rho d\theta d\varphi$$

**Figure 1:**
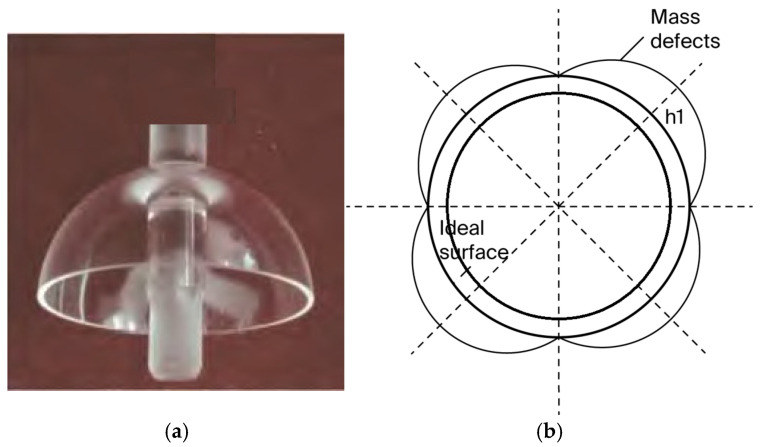
Schematic diagram of the resonator’s main mass defect. (**a**) Fused silica hemispherical shell resonator. (**b**) Fourth harmonic mass defects of hemispherical resonator.

Formula (8) reveals that the circumferential non-uniformity of the resonator’s density and wall thickness is the direct cause of frequency splitting. Let n = 2, which means the resonator vibrates in a second-order mode. The h and ρ in Formula (8) can be transformed into $h\rho =\rho_{0}h_{0}\left[m_{t}\left(\theta \right)\right]=\rho_{0}h_{0}\left(1+m_{1}\sin\frac{\theta }{2}+m_{2}\left|\sin\theta \right|+m_{3}\left|\sin\frac{3\theta }{2}\right|+m_{4}\left|\sin2\theta \right|+\ldots \right)$, where $\rho_{0}$ is the ideal density of the resonator, $h_{0}$ is the ideal wall thickness, and $m_{t}\left(\theta \right)$ is the mass defect function. Studies indicate that the frequency split caused by mass defects in the form of the fourth harmonic is significant compared to that caused by other harmonic forms, which can be neglected. Thus, this study primarily focuses on the frequency split due to mass defects in the fourth harmonic form and

(9) $$h\rho \approx \rho_{0}h_{0}\left(1+m_{4}\left|\sin2\theta \right|\right)=\rho_{0}\left(h_{0}+h_{1}\left|\sin2\theta \right|\right)=\rho_{0}h_{t}$$

where $h_{1}$ is the thickest thickness of the fourth harmonic mass defect and $h_{t}$ is the wall thickness of the hemispherical shell. According to Formula (9), it can be determined that all mass defects can be equivalent to wall thickness defects, and a schematic diagram of the resonator’s mass defect in the form of the fourth harmonic is shown in Figure 1: 

For the convenience of calculation, let $E,\mu ,\rho_{0}$ and R be constants, $\varphi_{F}=\pi /2$, $\varphi_{0}=0$. Substituting $h_{t}$ and Formula (9) into Formulas (6) and (8), the expression for the resonant frequency with a fourth harmonic mass defect is derived as follows:

(10) $$\omega_{4}=\sqrt{\frac{K_{1}}{m_{1}}}$$

(11) $$K_{1}=\frac{6E}{R^{4}\left(1+\mu \right)}\int_{0}^{\frac{\pi }{2}}\frac{\tan^{4}\frac{\varphi }{2}}{\sin^{3}\varphi }d\varphi \int_{0}^{2\pi }{\left(h_{0}+h_{1}\left|\sin2\theta \right|\right)}^{3}d\theta$$

(12) $$m_{1}=\rho_{0}m_{11}\int_{0}^{2\pi }\left(h_{0}+h_{1}\left|\sin2\theta \right|\right)d\theta +\frac{\rho_{0}}{2}\cos4\Psi m_{12}\int_{0}^{2\pi }\cos4\theta \left(h_{0}+h_{1}\left|\sin2\theta \right|\right)d\theta$$

where $m_{11}=\int_{0}^{\frac{\pi }{2}}\left[sin^{2}\varphi +\frac{1}{2}{\left(2+\cos\varphi \right)}^{2}\right]\tan^{4}\frac{\varphi }{2}\sin\varphi d\varphi =0.7648$

$$m_{12}=\int_{0}^{\frac{\pi }{2}}{\left(2+\cos\varphi \right)}^{2}\tan^{4}\frac{\varphi }{2}\sin\varphi d\varphi =1.1059$$

After simplification, the following can be obtained:

(13) $$K_{1}=\frac{7E}{4R^{4}\left(1+\mu \right)}\left(2\pi h_{0}^{3}+12h_{0}^{2}h_{1}+3\pi h_{0}h_{1}^{2}+\frac{8}{3}h_{1}^{3}\right)$$

(14) $$m_{1}=m_{21}+m_{22}\cos4\Psi =\rho_{0}\left(10\ln2-\frac{37}{6}\right)\left(4h_{1}+2\pi h_{0}\right)-0.7373\rho_{0}h_{1}\cos4\Psi$$

From Formula (14), it can be obtained that the position of the resonator’s heavy axis coincides with the thickest point of the fourth harmonic mass defect and the frequency split Δω is

(15) $$\Delta \omega =\omega_{l}-\omega_{h}$$

(16) $$\omega_{l}=\sqrt{\frac{7E}{4\rho_{0}R^{4}\left(1+\mu \right)}\frac{\left(2\pi h_{0}^{3}+12h_{0}^{2}h_{1}+3\pi h_{0}h_{1}^{2}+\frac{8}{3}h_{1}^{3}\right)}{\left(10\ln2-\frac{37}{6}\right)\left(4h_{1}+2\pi h_{0}\right)-0.7373h_{1}}}$$

(17) $$\omega_{h}=\sqrt{\frac{7E}{4\rho_{0}R^{4}\left(1+\mu \right)}\frac{\left(2\pi h_{0}^{3}+12h_{0}^{2}h_{1}+3\pi h_{0}h_{1}^{2}+\frac{8}{3}h_{1}^{3}\right)}{\left(10\ln2-\frac{37}{6}\right)\left(4h_{1}+2\pi h_{0}\right)+0.7373h_{1}}}$$

The physical and geometrical parameters of the resonator are listed in Table 1.

**Figure 2:**
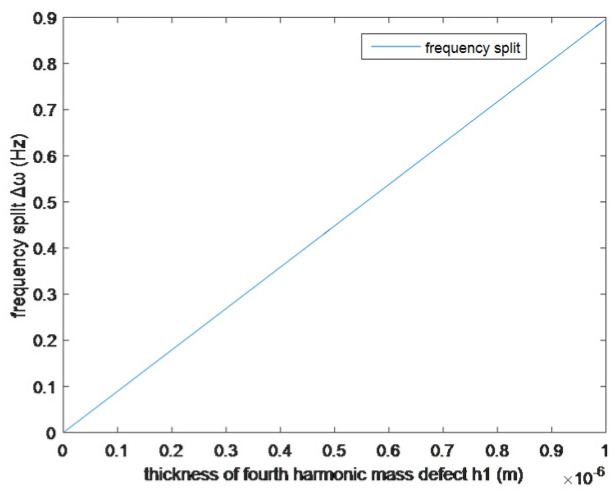
Effect of wall thickness of fourth harmonic mass defect on frequency split.

The simulation of the relationship between the frequency split and mass defect wall thickness is shown in Figure 2.

In this example, only when the processing accuracy is less than 0.55 nm can the frequency split of the resonator meet the navigation requirements. Therefore, it is impossible to obtain high-precision resonators through mechanical processing, and the simulation result also explains why it is necessary to perform the balancing process.

## 3. Influence of Ion Beam Balancing on Resonator Frequency

Ion beam balancing mainly requires two problems to be solved: where to balance and how deep to balance. The following research will focus on these two issues.

### 3.1. Balancing Theory and Model

**Figure 3:**
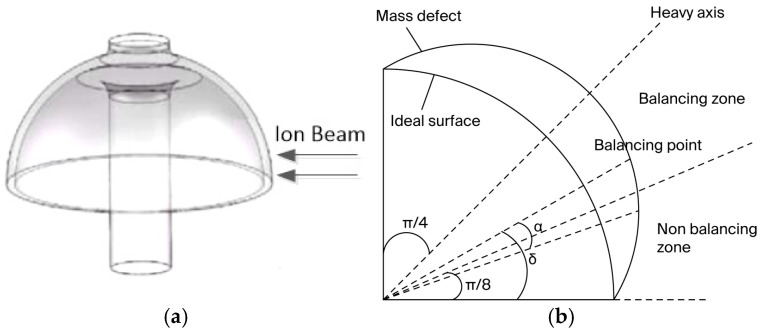
Balancing position of the resonator. (**a**) Appearance of ion beam balancing. (**b**) Top view of ion beam balancing.

Taking a quarter of the fourth harmonic mass defect of the resonator as an example, and as shown in Figure 3, the balancing position is the circumferential angle δ, the balancing width is 2α of the circumferential angle, the balancing height is β of the generatrix angle, and the balancing function, which takes the balancing axis as the symmetry axis, is $h_{t}$. For the convenience of the calculation, the height position of the generatrix angle is set to $\left(\pi /2-\beta ,\pi /2\right)$.

The influence of $h_{t}$ on the resonator frequency is $K_{b1}$ and $m_{b1}$:

(18) $$K_{b1}=\frac{6E}{R^{4}\left(1+\mu \right)}\int_{\frac{\pi }{2}-\beta }^{\frac{\pi }{2}}\frac{\tan^{4}\frac{\varphi }{2}}{\sin^{3}\varphi }d\varphi \int_{\delta -\alpha }^{\delta +\alpha }\left[{\left(h_{0}+h_{1}\sin2\theta +h_{t}\right)}^{3}-{\left(h_{0}+h_{1}\sin2\theta \right)}^{3}\right]d\theta$$

(19) $$m_{b1}=m_{bq11}+m_{bq12}\cos4\Psi +m_{bq13}\sin4\Psi$$

where $m_{bq11}=\rho_{0}\int_{\frac{\pi }{2}-\beta }^{\frac{\pi }{2}}\left[\sin^{2}\varphi +\frac{1}{2}{\left(2+\cos\varphi \right)}^{2}\right]\tan^{4}\frac{\varphi }{2}\sin\varphi d\varphi \int_{\delta -\alpha }^{\delta +\alpha }\left(h_{t}\right)d\theta$

$$m_{bq12}=\frac{\rho_{0}}{2}\int_{\frac{\pi }{2}-\beta }^{\frac{\pi }{2}}{\left(2+\cos\varphi \right)}^{2}\tan^{4}\frac{\varphi }{2}\sin\varphi d\varphi \int_{\delta -\alpha }^{\delta +\alpha }\cos4\theta \left(h_{t}\right)d\theta$$

$$m_{bq13}=\frac{\rho_{0}}{2}\int_{\frac{\pi }{2}-\beta }^{\frac{\pi }{2}}{\left(2+\cos\varphi \right)}^{2}\tan^{4}\frac{\varphi }{2}\sin\varphi d\varphi \int_{\delta -\alpha }^{\delta +\alpha }\sin4\theta \left(h_{t}\right)d\theta$$

Increasing δ from 0 to π/2, $m_{bq3}$ is only equal to 0 when δ=0,π/4,π/2. However, balancing on the light axis will only increase the frequency split, so a balancing method for the fourth harmonic quality defect of the resonator is to balance on the heavy axis (δ=π/4).

Through further analysis, the following was discovered:

(20) $$\int_{\frac{\pi }{8}-\alpha }^{\frac{\pi }{8}+\alpha }\cos4\theta \left(h_{t}\right)d\theta =\int_{\frac{3\pi }{8}-\alpha }^{\frac{3\pi }{8}+\alpha }\cos4\theta \left(h_{t}\right)d\theta =0$$

This indicates that balancing within the range of π/8 on both sides of the light axis will increase frequency splitting and the balancing point, which can reduce the frequency splitting, this is shown only in the balancing zone in Figure 3.

After confirming the balancing point, the next issue is how deep the resonator needs to balance. Considering the entire balancing function of the resonator, the frequency of the resonator is

(21) $$\omega_{4b}=\sqrt{\frac{K_{1}+K_{b1}+K_{b2}+K_{b3}+K_{b4}}{m_{1}+m_{b1}+m_{b2}+m_{b3}+m_{b4}}}$$

where

(22) $$\left\{\begin{array}{c} \begin{array}{c} K_{b2}=K_{02}\int_{\frac{3\pi }{4}-\alpha }^{\frac{3\pi }{4}+\alpha }\left[{\left(h_{0}-h_{1}\sin2\theta +h_{t}\right)}^{3}-{\left(h_{0}-h_{1}\sin2\theta \right)}^{3}\right]d\theta \\ K_{b3}=K_{02}\int_{\frac{5\pi }{4}-\alpha }^{\frac{5\pi }{4}+\alpha }\left[{\left(h_{0}+h_{1}\sin2\theta +h_{t}\right)}^{3}-{\left(h_{0}+h_{1}\sin2\theta \right)}^{3}\right]d\theta \\ K_{b4}=K_{02}\int_{\frac{7\pi }{4}-\alpha }^{\frac{7\pi }{4}+\alpha }\left[{\left(h_{0}-h_{1}\sin2\theta +h_{t}\right)}^{3}-{\left(h_{0}-h_{1}\sin2\theta \right)}^{3}\right]d\theta \end{array} \end{array}\right.$$

(23) $$\left\{\begin{array}{c} \begin{array}{c} m_{b2}=m_{21}\int_{\frac{3\pi }{4}-\alpha }^{\frac{3\pi }{4}+\alpha }\left(h_{t}\right)d\theta +m_{22}\int_{\frac{3\pi }{4}-\alpha }^{\frac{3\pi }{4}+\alpha }\cos4\theta \left(h_{t}\right)d\theta cos4\Psi \\ m_{b3}=m_{21}\int_{\frac{5\pi }{4}-\alpha }^{\frac{5\pi }{4}+\alpha }\left(h_{t}\right)d\theta +m_{22}\int_{\frac{5\pi }{4}-\alpha }^{\frac{5\pi }{4}+\alpha }\cos4\theta \left(h_{t}\right)d\theta cos4\Psi \\ m_{b4}=m_{21}\int_{\frac{7\pi }{4}-\alpha }^{\frac{7\pi }{4}+\alpha }\left(h_{t}\right)d\theta +m_{22}\int_{\frac{7\pi }{4}-\alpha }^{\frac{7\pi }{4}+\alpha }\cos4\theta \left(h_{t}\right)d\theta cos4\Psi \end{array} \end{array}\right.$$

(24) $$K_{02}=\frac{6E}{R^{4}\left(1+\mu \right)}\int_{\frac{\pi }{2}-\beta }^{\frac{\pi }{2}}\frac{\tan^{4}\frac{\varphi }{2}}{\sin^{3}\varphi }d\varphi$$

(25) $$m_{21}=\rho_{0}\int_{\frac{\pi }{2}-\beta }^{\frac{\pi }{2}}\left[\sin^{2}\varphi +\frac{1}{2}{\left(2+\cos\varphi \right)}^{2}\right]\tan^{4}\frac{\varphi }{2}\sin\varphi d\varphi$$

(26) $$m_{22}=\frac{\rho_{0}}{2}\int_{\frac{\pi }{2}-\beta }^{\frac{\pi }{2}}{\left(2+\cos\varphi \right)}^{2}\tan^{4}\frac{\varphi }{2}\sin\varphi d\varphi$$

The coefficient of cos4Ψ in Formula (21) is

(27) $$m_{c0}=-0.7373\rho_{0}h_{1}+4m_{22}\int_{\frac{\pi }{4}-\alpha }^{\frac{\pi }{4}+\alpha }\cos4\theta \left(h_{t}\right)d\theta$$

Let $h_{t}=h_{b}f\left(\theta \right)$, in which $h_{b}$ is the balancing depth, and $f\left(\theta \right)$ is the balancing function related to the circumferential angle θ. When $m_{c}=0$, Δω=0, and the balancing depth is

(28) $$h_{b0}=\frac{0.7373\rho_{0}h_{1}}{4m_{22}\int_{\frac{\pi }{4}-\alpha }^{\frac{\pi }{4}+\alpha }\cos4\theta f\left(\theta \right)d\theta }$$

To more directly observe the relationship between the balancing depth and frequency split, the model simulation parameters are shown in Table 1 and Table 2.

The resonant frequency with heavy-axis balancing is

(29) $$\omega_{4b}=A_{0}\sqrt{\frac{K_{1}+K_{b1}+K_{b2}+K_{b3}+K_{b4}}{b_{0}h_{0}+b_{1}h_{1}+b_{2}h_{2}+\cos4\Psi \left(b_{3}h_{1}+b_{4}h_{2}\right)}}$$

where $A_{0}=\sqrt{\frac{6E}{\rho R^{4}\left(1+\mu \right)}}$

$$K_{1}+K_{b1}+K_{b2}+K_{b3}+K_{b4}=a_{0}h_{0}^{3}+a_{1}h_{0}^{2}h_{1}+a_{2}h_{0}^{2}h_{2}+a_{3}h_{0}h_{1}^{2}\;\;\;\;\;\;\;\;\;\;\;\;\;\;\;\;\;\;\;\;\;+a_{4}h_{0}h_{1}h_{2}+a_{5}h_{0}h_{2}^{2}+a_{6}h_{1}^{3}+a_{7}h_{1}^{2}h_{2}+a_{8}h_{1}h_{2}^{2}+a_{9}h_{2}^{3}$$

and the parameters in $\omega_{4b}$ are shown in Table 3.

**Figure 4:**
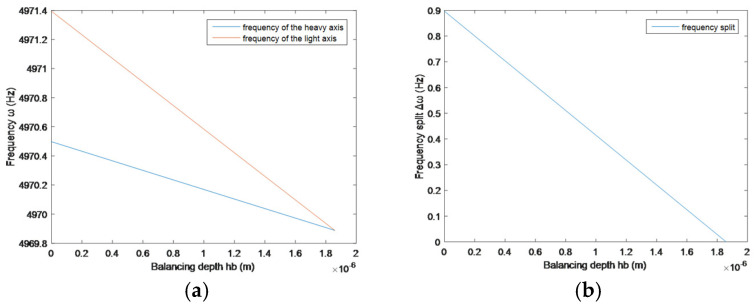
Variation in frequency characteristics during the balancing process. (**a**) Variation in the eigenfrequency with balancing depth. (**b**) Variation in the frequency split with balancing depth.

When $h_{b0}=1.858h_{1}=1.858\;\mu m$, the frequency split of the resonator is balanced to 0 Hz. The variation in frequency characteristics is shown in Figure 4:

During the balancing process, the heavy axis of the resonator will not drift. When the balancing depth exceeds $h_{b0}$, the positions of the light and heavy axes of the resonator will be swapped, and it is necessary to balance on the new heavy axis.

### 3.2. Impact of Balancing Errors

The actual balancing process of the resonator cannot be consistent with the theoretical balancing because of two main sources of error: one is the uneven balancing depth on the four heavy axes affected by the stability of the ion beam, and the other is the misalignment of the balancing position affected by the accuracy of the turntable and the identification accuracy of the heavy axis.

#### 3.2.1. Impact of the Balancing Depth Error on the Heavy Axis

When the balancing depths on the heavy axes are inconsistent due to the stability limitations of the ion beam, the balancing functions on the heavy axis are

(30) $$\left\{\begin{array}{c} h_{t1}=h_{b1}f\left(\theta \right)\\ h_{t2}=h_{b2}f\left(\theta \right)\\ h_{t3}=h_{b3}f\left(\theta \right)\\ h_{t4}=h_{b4}f\left(\theta \right) \end{array}\right.$$

where $h_{b1},h_{b2},h_{b3},h_{b4}$ are the balancing depths on the respective heavy axes, and the coefficient of cos4Ψ in Formula (21) is

(31) $$m_{c01}=-0.7373\rho_{0}h_{1}+\left(h_{b1}+h_{b2}+h_{b3}+h_{b4}\right)m_{22}\int_{\frac{\pi }{4}-\alpha }^{\frac{\pi }{4}+\alpha }\cos4\theta f\left(\theta \right)d\theta$$

Comparing Formula (27) and Formula (31), it can be concluded that as long as the balancing depths satisfy

(32) $$4h_{b0}=h_{b1}+h_{b2}+h_{b3}+h_{b4}$$

the frequency split can be balanced to 0 Hz. Furthermore, when the frequency split of the fourth harmonic mass defect of the resonator has been balanced very low, the balancing can be concentrated on one heavy axis, and the frequency split can also be balanced to 0 Hz.

#### 3.2.2. Impact of the Alignment Error

Assuming that the balancing points are misaligned in a single direction and the balancing alignment angle is τ, the frequency of the resonator is

(33) $$\omega_{4b\tau }=\sqrt{\frac{K_{1}+K_{b\tau 1}+K_{b\tau 2}+K_{b\tau 3}+K_{b\tau 4}}{m_{1}+m_{b\tau 1}+m_{b\tau 2}+m_{b\tau 3}+m_{b\tau 4}}}$$

where

(34) $$\left\{\begin{array}{l} K_{b\tau 1}=K_{02}\int_{\frac{\pi }{4}-\alpha -\tau }^{\frac{\pi }{4}+\alpha -\tau }\left[{\left(h_{0}+\sin2\theta +h_{t\tau }\right)}^{3}-{\left(h_{0}+h_{1}\sin2\theta \right)}^{3}\right]d\theta \\ K_{b\tau 2}=K_{02}\int_{\frac{3\pi }{4}-\alpha -\tau }^{\frac{3\pi }{4}+\alpha -\tau }\left[{\left(h_{0}-\sin2\theta +h_{t\tau }\right)}^{3}-{\left(h_{0}-h_{1}\sin2\theta \right)}^{3}\right]d\theta \\ K_{b\tau 3}=K_{02}\int_{\frac{5\pi }{4}-\alpha -\tau }^{\frac{5\pi }{4}+\alpha -\tau }\left[{\left(h_{0}+h_{1}\sin2\theta +h_{t\tau }\right)}^{3}-{\left(h_{0}+h_{1}\sin2\theta \right)}^{3}\right]d\theta \\ K_{b\tau 4}=K_{02}\int_{\frac{7\pi }{4}-\alpha -\tau }^{\frac{7\pi }{4}+\alpha -\tau }\left[{\left(h_{0}-\sin2\theta +h_{t\tau }\right)}^{3}-{\left(h_{0}-h_{1}\sin2\theta \right)}^{3}\right]d\theta \end{array}\right.$$

(35) $$\left\{\begin{array}{l} m_{b\tau 1}=m_{21}\int_{\frac{\pi }{4}-\alpha -\tau }^{\frac{\pi }{4}+\alpha -\tau }\left(h_{t\tau }\right)d\theta +m_{22}\int_{\frac{\pi }{4}-\alpha -\tau }^{\frac{\pi }{4}+\alpha -\tau }\cos4\theta \left(h_{t\tau }\right)d\theta \cos4\Psi +m_{22}\int_{\frac{\pi }{4}-\alpha -\tau }^{\frac{\pi }{4}+\alpha -\tau }\sin4\theta \left(h_{t\tau }\right)d\theta \sin4\Psi \\ m_{b\tau 2}=m_{21}\int_{\frac{3\pi }{4}-\alpha -\tau }^{\frac{3\pi }{4}+\alpha -\tau }\left(h_{t\tau }\right)d\theta +m_{22}\int_{\frac{3\pi }{4}-\alpha -\tau }^{\frac{3\pi }{4}+\alpha -\tau }\cos4\theta \left(h_{t\tau }\right)d\theta \cos4\Psi +m_{22}\int_{\frac{3\pi }{4}-\alpha -\tau }^{\frac{3\pi }{4}+\alpha -\tau }\sin4\theta \left(h_{t\tau }\right)d\theta \sin4\Psi \\ m_{b\tau 3}=m_{21}\int_{\frac{5\pi }{4}-\alpha -\tau }^{\frac{5\pi }{4}+\alpha -\tau }\left(h_{t\tau }\right)d\theta +m_{22}\int_{\frac{5\pi }{4}-\alpha -\tau }^{\frac{5\pi }{4}+\alpha -\tau }\cos4\theta \left(h_{t\tau }\right)d\theta \cos4\Psi +m_{22}\int_{\frac{5\pi }{4}-\alpha -\tau }^{\frac{5\pi }{4}+\alpha -\tau }\sin4\theta \left(h_{t\tau }\right)d\theta \sin4\Psi \\ m_{b\tau 4}=m_{21}\int_{\frac{7\pi }{4}-\alpha -\tau }^{\frac{7\pi }{4}+\alpha -\tau }\left(h_{t\tau }\right)d\theta +m_{22}\int_{\frac{7\pi }{4}-\alpha -\tau }^{\frac{7\pi }{4}+\alpha -\tau }\cos4\theta \left(h_{t\tau }\right)d\theta \cos4\Psi +m_{22}\int_{\frac{7\pi }{4}-\alpha -\tau }^{\frac{7\pi }{4}+\alpha -\tau }\sin4\theta \left(h_{t\tau }\right)d\theta \sin4\Psi \end{array}\right.$$

where $h_{t\tau }=h_{b\tau }f\left(\theta \right)$, and $h_{b\tau }$ is the balancing depth with the alignment angle.

After transformation, $\left(m_{1}+m_{b\tau 1}+m_{b\tau 2}+m_{b\tau 3}+m_{b\tau 4}\right)$ becomes

(36) $$m_{1}+m_{b\tau 1}+m_{b\tau 2}+m_{b\tau 3}+m_{b\tau 4}=m_{\tau 0}+A_{\tau 0}\cos\left(4\Psi +\theta_{\tau }\right)$$

in which

(37) $$m_{\tau 0}=\rho_{0}m_{11}\int_{0}^{2\pi }\left(h_{0}+h_{1}\left|\sin2\theta \right|\right)d\theta +4m_{21}\int_{\frac{\pi }{4}-\alpha -\tau }^{\frac{\pi }{4}+\alpha -\tau }\left(h_{t\tau }\right)d\theta$$

(38) $$\tan(\theta_{\tau })=\frac{4m_{22}\int_{\frac{\pi }{4}-\alpha -\tau }^{\frac{\pi }{4}+\alpha -\tau }\sin4\theta \left(h_{t\tau }\right)d\theta }{-0.7373\rho_{0}h_{1}+4m_{22}\int_{\frac{\pi }{4}-\alpha -\tau }^{\frac{\pi }{4}+\alpha -\tau }\cos4\theta \left(h_{t\tau }\right)d\theta }$$

(39) $$A_{\tau 0}=\left\{{\left[-0.7373\rho_{0}h_{1}+4m_{22}\int_{\frac{\pi }{4}-\alpha -\tau }^{\frac{\pi }{4}+\alpha -\tau }\cos4\theta h_{b\tau }f\left(\theta \right)d\theta \right]}^{2}\right.\;{\left.+{\left(4m_{22}\int_{\frac{\pi }{4}-\alpha -\tau }^{\frac{\pi }{4}+\alpha -\tau }\sin4\theta h_{b\tau }f\left(\theta \right)d\theta \right)}^{2}\right\}}^{\frac{1}{2}}=\sqrt{{\left(A_{\tau 1}h_{b\tau }-A_{\tau 2}\right)}^{2}+A_{\tau 3}}>0$$

Formula (39) states that the frequency split of the fourth harmonic mass defect of the resonator cannot be balanced to 0 Hz on the heavy axis with misalignment error.

**Figure 5:**
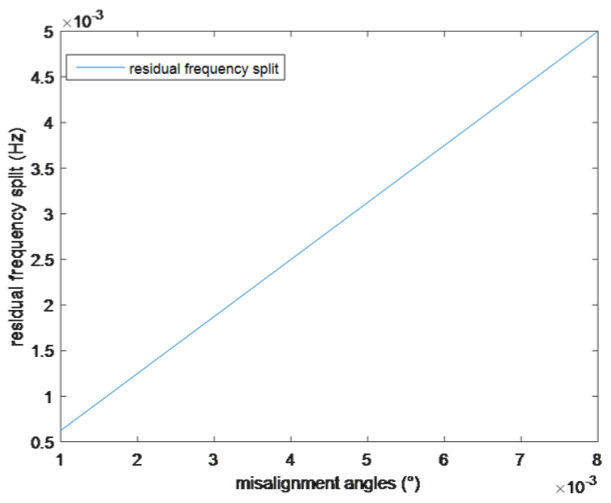
Relationship between the residual frequency split and the misalignment angles.

Using the parameters in Table 1 and Table 2, and setting τ from 0.001° to 0.008°, the frequency characteristics have been simulated. The balancing depths of the resonator with different misalignment angles are approximately equal to the ideal balancing depth $1.858h_{1}=1.858\;\mu m$, but it is difficult to distinguish the frequency variation in the balancing processes with a misalignment angle from the ideal balancing processes through diagrams. The relationship between the residual frequency split and the misalignment angles is shown in Figure 5.

The simulation results show that for every 0.001° increase in τ, the residual frequency split increases by 0.0000625 Hz, and when τ=0.008°, the resonator cannot meet the navigation requirements. In the actual balancing system, the position precision of the turntable equipment can reach 0.001°, but it is hard for the identification precision of the heavy axis to reach such a high precision. For the parameter identification system used in this article, the identification error of the heavy axis is less than 0.05°, and the frequency split may rebound when it is balanced to 0.00313 Hz.

**Figure 6:**
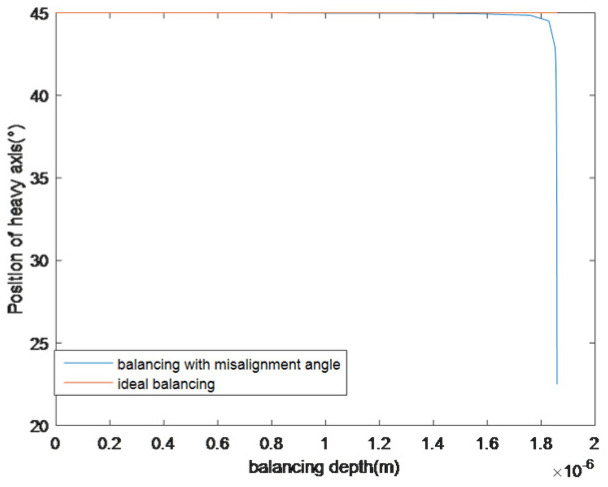
Position of the heavy axis in balancing process.

Assuming τ=0.008°, the position of the heavy axis has been simulated and the result is shown in Figure 6.

For ideal balancing, the heavy axis of the resonator will not move. For balancing processes with a misalignment angle, the heavy axis of the resonator will slide to a new position at the end of the balancing process. Specifically, in this simulation example, the position of the heavy axis after balancing is 22.508°. When the balancing misalignment is not reduced and a second balancing is performed on the new heavy axis, the new balancing position is exactly in the non-balancing zone in Figure 3 and the frequency split will increase. Therefore, when the misalignment angle cannot be reduced, the resonator manufacturer is required to improve the accuracy. When the wall thickness of the fourth harmonic mass defect of the resonator is 0.1 μm, in this simulation example, the frequency split is 0.0896 Hz and the balancing depth is 0.186 μm. The frequency split can be reduced to 0.000494 Hz, when τ=0.079°. 

## 4. Experimental Results of Ion Beam Balancing and Discussion

**Figure 7:**
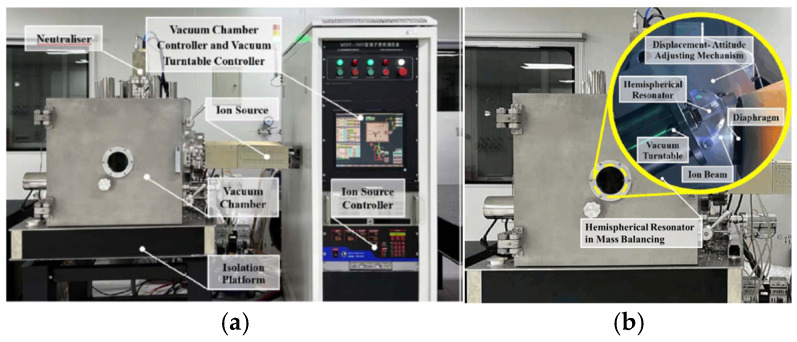
Ion beam etching system. (**a**) Overall composition of ion beam etching system. (**b**) Hemispherical resonator in mass balancing of ion beam.

In order to verify the correctness of the theoretical model and the superiority of the mass balancing process, a mass defect balancing experiment is carried out. The ion beam balancing system is shown in Figure 7 and the steps of the balancing process are as follows:

(1) Determine the heavy axis and frequency split of the resonator by using the measurement system of vibration parameters.
(2) Estimate the balancing depth of the resonator.
(3) Generate the ion beam, which etches the outer hemispherical surface through the diaphragm aperture.
(4) Check whether the frequency split meets the requirement.
(5) If the test results meet the requirements, stop balancing and remove the resonator. Otherwise, measure the resonator frequency split again and return to step 2.

**Figure 8:**
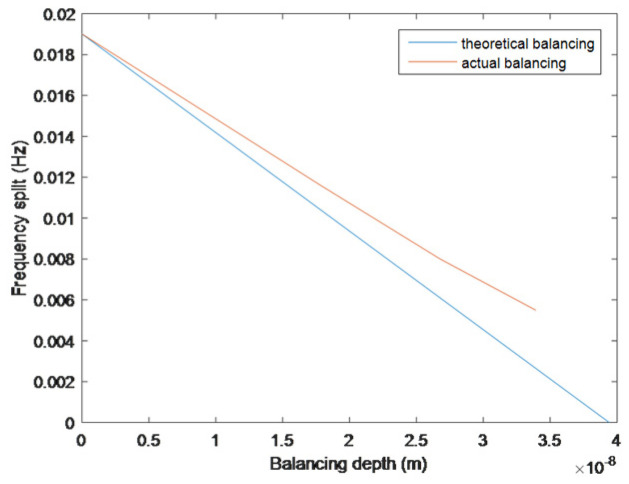
Balancing process of resonator #1.

For the experiment, resonator #1 was used for balancing. The frequency split was 0.019 Hz, which could be fitted to a fourth harmonic mass defect wall thickness of 0.0212 μm, and the quality factor of the resonator is $1.47\times 10^{7}$. Using the heavy-axis balancing method, the process was conducted three times in total. The first balancing time was 600 s, and the frequency split reduced to 0.0116 Hz. The second balancing time was 300 s, and the frequency split reduced to 0.008 Hz. The last balancing time was 240 s, and the frequency split was reduced to 0.0055 Hz. The trimming rate is 0.8 nm/min. The balancing process is shown in Figure 8.

As shown in Figure 8, in the later stage of balancing, the actual balancing curve gradually deviates from the theoretical curve. This result is not only affected by factors such as ion beam accuracy and alignment accuracy, but also by the impact of other harmonic and non-harmonic quality defects on the resonator. The frequency split caused by these quality defects can be ignored numerically, but the presence of these quality defects can affect the position of the heavy axis and the balancing effect of frequency splitting.

The quality factor of the resonator after balancing is $9.5\times 10^{6}$. The main task of the ion bean balancing process is to make the frequency split of the resonator meet the requirement. The change in the quality factor can be temporarily ignored in this process, and adjusted in other processes such as chemical etching.

**Figure 9:**
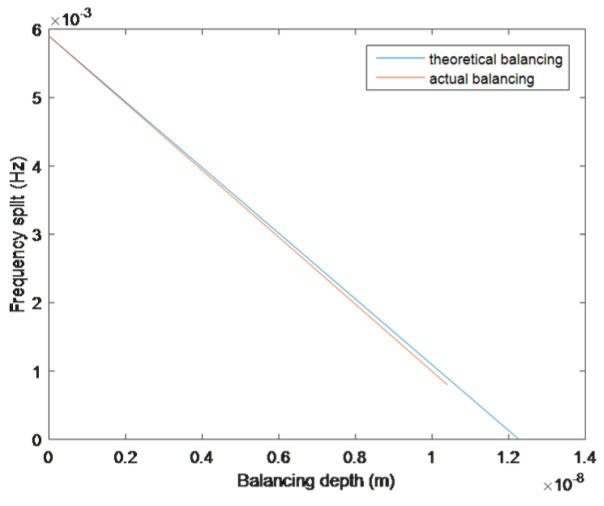
Balancing process of resonator #2.

Another resonator, resonator #2, was chosen to verify the effectiveness of the theory again. After testing, the frequency split was 0.0059 Hz, which was fitted to a fourth harmonic mass defect wall thickness of 0.0066 μm and the quality factor of the resonator was $8.01\times 10^{6}$. Using the heavy-axis balancing method, the duration of the balancing process was 300 s and the frequency split of the resonator was reduced to 0.0008 Hz. The trimming rate was 1nm/min and the quality factor after balancing was $6.22\times 10^{6}$.The balancing process is shown in Figure 9. 

## 5. Conclusions

This article establishes a model for the vibration frequency, mass defect, and balancing function of a hemispherical resonator based on the energy equation. This model is applied to analyze the influence of the ion beam balancing process on the resonant frequency and frequency split. Firstly, by equating mass defects to fourth harmonic wall thickness defects in the resonator, the relationship between the frequency split and resonator defect wall thickness is established.

Then, the balancing function and the balancing errors are introduced into the frequency formula. After calculation and analysis, it can be seen that for the second-order vibration mode, the balancing point of the fourth harmonic mass defect of the resonator coincides with the heavy axis of the resonator. Theoretically, the frequency split can be balanced to 0 Hz, and the position of the heavy axis remains unchanged. In the area of π/8 on both sides of the heavy axis, balancing can also reduce the frequency split, but in the area of π/8 on both sides of the light axis, balancing will only increase the frequency split. For the balancing errors, the influence of the balancing depth error on the frequency split is less than that of the alignment error. When the alignment error is not considered, it is not strictly required for the balancing depth on the heavy axes to be consistent. As long as the sum of the four balancing depths is equal to the sum of the ideal balancing depths, the resonator can be balanced to 0 Hz. When there is an alignment error and the error remains unchanged, the resonator cannot be completely balanced to 0 Hz. The residual frequency split is related to the misalignment angle. 

Finally, the effectiveness of the theory was verified through experiments. The experimental results show that when the frequency split of the resonator is high, the precision requirement for initial alignment of the heavy axis is correspondingly increased. When the frequency split is low, the requirement for the initial alignment can be reduced, and the frequency split can be balanced to better than 0.001 Hz.

## Figures and Tables

**Table 1:** Physical and geometrical parameters.

Variable	Parameter	Simulation Value
*E*	elastic modulus	$7.67\times 10^{10}\;\mathrm{Pa}$
*R*	mid-surface radius	0.015 m
$\rho_{0}$	ideal density	$2200\;{\mathrm{kg}/m}^{3}$
μ	Poisson’s ratio	0.17
$h_{0}$	ideal wall thickness	0.85 mm

**Table 2:** Balancing parameters.

Variable	Parameter	Simulation Value
α	balancing width	π/6
β	balancing height	π/9
$h_{t}$	balancing function	$-h_{b}\cos8\theta$

**Table 3:** Parameters in *ω*_4*b*_.

Variable	Simulation Value	Variable	Simulation Value
*a*_0_	4.856	*a*_8_	0.456
*a*_1_	9.275	*a*_9_	−0.130
*a*_2_	−0.586	*b*_0_	4.805
*a*_3_	7.284	*b*_1_	3.059
*a*_4_	−1.155	*b*_2_	−0.601
*a*_5_	0.460	*b3*	−0.737
*a*_6_	2.061	*b*_4_	0.397
*a*_7_	−0.569		

## Data Availability

Date will be made available on request. Date will be made available from the corresponding author upon reasonable request.
